# Automated 3D morphometric difference biomarker for abnormal ventricular morphology

**DOI:** 10.1186/1532-429X-15-S1-E35

**Published:** 2013-01-30

**Authors:** Prahlad G Menon, Robert W Biederman

**Affiliations:** 1 Biomedical Engineering, Carnegie Mellon University, Pittsburgh, PA, USA; 2 Cardiac MRI, Allegheny General Hospital, Pittsburgh, PA, USA

## Background

Left-ventricular (LV) shape remodeling has known association with cardiac pathophysiology and dysfunction. While a prolate spheroid shape is vital for optimal cardiac function, LV dilatation and high sphericity is characteristic of cardiomyopathy. Early quantification of abnormal LV morphology may potentially guide clinical decisions to check disease progression. However a quantitative biomarker of 3D LV morphometry is found wanting in routine clinical practice as an upgrade to the rudimentary sphericity index.

## Methods

Spherical harmonics (SPHARM) shape descriptors were sought to quantify patient-specific LV shape. Morphometric variation from a normal LV shape was studied with application to diagnostic identification of cardiomyopathy. Cardiac MR (CMR) was employed for anatomical examination and end-diastolic LV endocardial surface models were segmented from short-axis SSFP cine scans of 11 pediatric subjects between the ages 2 and 17. Surface models were individually registered by rigid transformation and scaling to match the LV base diameter of a reference 17 year old normal LV that approximated well to a prolate spheroid. Two characteristic morphometric difference percentages (DP) were computed as a percentage of normal LV length for each case with respect to the reference normal model i.e. maximum DP (MDP) and average DP (ADP). A 16 patient cohort was used for training, comprising 10 simulated normals (ADP < 3.5 % & MDP < 13%) and 6 abnormal cases including 2 with LV hypertrophy (LVH), 3 with arrythmogenic right ventricular dysplasia (ADVD), 1 post myocardial infarction (MI). Rule-based decision trees were prepared to predict a response function defining normal vs. pathological cases using the new MDP and ADP predictors simultaneously with the conventional LV sphericity index as a third predictor, through a 3D generative clustering approach. Classification accuracy was evaluated with a 6 patient test cohort comprising 3 ARVDs, 2 LVH and 2 normal subjects.

## Results

The underlying principle of this LV-base normalized diffeomorphic analysis is that LV remodeling is accompanied by heightened sphericity. This assumption was verified by the existence of positive Spearman rank correlations for sphericity with ADP (0.41) and MDP (0.28). 100% test classification accuracy was obtained using a simultaneous thresholds for MDP (>13.9%), ADP (>3.5%) and sphericity (>0.7). ARVD patients were all identified as pathologies based upon LV shape alone. Further, strong correlation (0.94) was observed between LV sphericity and age for ARVD patients.

## Conclusions

This pilot study suggests DP is a valuable biomarker in identifying cardiomyopathy. Strong correlation between sphericity and ARVD patient age combined with the excellent classification of ARVD as a cardiomyopathy by LV shape alone supports possible left-sided involvement in ARVD. Analysis with a larger cohort comprising more normal controls is warranted to validate this hypothesis.

## Funding

No funding sources to disclose.

**Table 1:** 

Sl no	Diagnosis	Sphericity	Age	MDP % of normal LV length	ADP % of normal LV length	Rank, Sphericity (low to high)	Rank, MDP (low to high)	Rank, ADP (low to high)
1	ARVD	0.600	2	14.408	4.307	2	5	8
2	ARVD	0.625	6	14.826	4.083	5	6	7
3	ARVD	0.643	7	22.460	5.648	6	12	12
4	LVH	0.672	8	17.546	4.789	8	10	9
5	ARVD	0.703	9	11.82	5.464	10	2	11
6	ARVD	0.726	11	11.941	3.731	11	3	6
7	NORMAL	0.616	13	13.860	3.496	3	4	5
8	ARVD	0.703	15	15.282	2.879	9	8	2
9	LVH	0.625	16	15.930	2.953	4	9	3
10	MI	0.726	16	18.913	5.099	12	11	10
11	NORMAL *	0.584	17	0.000	0.000	1	1	1
12	LVH	0.662	17	15.253	3.356	7	7	4

		Spearman Correlation with Sphericity	0.943	0.280	0.406			

* reference normal

**Figure 1:**
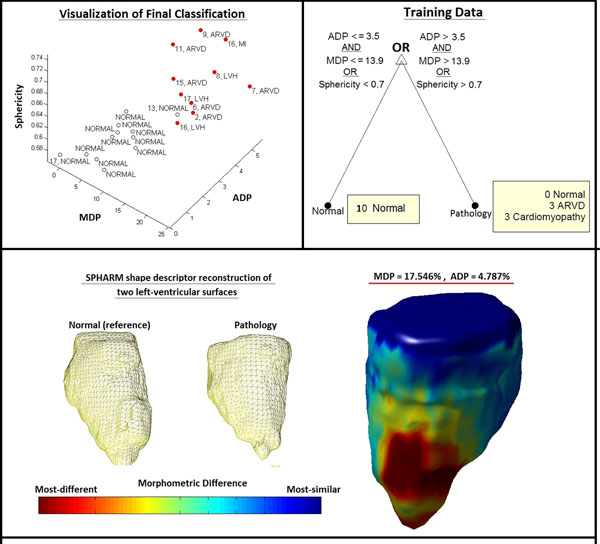
Top left: Generative classification result in a 3D space defined by sphericity, MDP and ADP (Red: pathology, Blue: normal). Each patient is identified by a label constituted of "Age, Diagnosis" . Top right: Decision tree produced from the training data set, using either MDP or ADP. Bottom: 3D morphometric difference colormap mapped onto the surface of a pathological LV model. 40th degree SPHARM representations of a normal and a representative pathological LV endocardium surfaces are shown, as well.

